# United States Department of Agriculture nutrition assistance programs during the COVID-19 pandemic: A scoping review protocol

**DOI:** 10.1371/journal.pone.0288585

**Published:** 2023-07-19

**Authors:** Jessica Soldavini, Margaret Read, Lauren Clay

**Affiliations:** 1 Center for Health Promotion and Disease Prevention and Department of Nutrition, Gillings School of Global Public Health, University of North Carolina at Chapel Hill, Chapel Hill, North Carolina, United States of America; 2 No Kid Hungry Campaign, Share Our Strength, Washington, DC, United States of America; 3 Department of Emergency Health Sciences, University of Maryland Baltimore County, Baltimore, Maryland, United States of America; Drexel University, UNITED STATES

## Abstract

**Objective:**

The goal of this scoping review is to examine the published research on federal nutrition assistance programs administered by the United States (U.S.) Department of Agriculture during the COVID-19 pandemic, in the U.S., U.S. territories, and tribal nations. The review will identify the scope of the available research and provide research and policy recommendations.

**Introduction:**

The COVID-19 pandemic made individuals more vulnerable to experiencing food insecurity. Federal nutrition assistance programs help to address food insecurity and have been rapidly adapting to meet food and nutrition needs among affected communities during the COVID-19 pandemic. It is important to understand the scope of the current research on this topic to help inform future research, practice, and policy recommendations.

**Inclusion criteria:**

This review will include studies focused on federal nutrition assistance programs administered by the U.S. Department of Agriculture during the COVID-19 pandemic. The scoping review will consider all primary research designs.

**Methods:**

Pubmed, CINHAL, Scopus, and Proquest’s Health Management databases will be used for the literature search. Only articles published in English since March 1, 2020 will be considered. Titles/abstracts followed by full-text articles will be reviewed to determine which articles meet the inclusion criteria and should be included in the review. Data will be extracted from each included article using a data extraction template in Covidence that will be developed by the study team. Data extracted will include information on key findings related to the review questions. At each step, two independent reviewers will be assigned to each article. Data will be summarized and presented in tables, charts, and narrative summary.

## Introduction

The Coronavirus Disease 2019 (COVID-19) pandemic is one of the largest public health emergencies the United States (U.S.) has faced. The U.S. Department of Health and Human services has determined this to be a public health emergency since January 27, 2020 [1] and the President of the U.S. issued a proclamation on March 13, 2020 declaring the COVID-19 pandemic to be a national emergency as of March 1, 2020 [2].

Food insecurity is also a serious public health concern in the U.S. that is associated with a variety of negative health consequences [3]. In 2020, 10.5% of U.S. households were food insecure [4]. The COVID-19 pandemic can make individuals more vulnerable to experiencing food insecurity [5]. The U.S. Department of Agriculture (USDA) administers a variety of federal food and nutrition assistance programs to help address food insecurity, such as the Supplemental Nutrition Assistance Program (SNAP); Child Nutrition Programs; and Special Supplemental Nutrition Program for Women, Infants, and Children (WIC) [6].

Since the pandemic began, federal nutrition assistance programs have had to adapt rapidly to meet food and nutrition needs among affected communities [7–9]. The Families First Coronavirus Response Act gave USDA the authority to issue nationwide waivers for regulations for federal nutrition programs [10]. Numerous waivers have been issued throughout the pandemic for programs such as SNAP, Child Nutrition Programs, and WIC to help make it more feasible to administer these programs given the context of the COVID-19 pandemic [11]. New programs such pandemic electronic benefit transfer (P-EBT), which provided EBT cards with benefits that families could use to purchase food, and Emergency Meals-to-You, which mailed shelf-stable meals to children in rural communities, have also been developed during the pandemic to help address food insecurity [11]. Many of the waivers, adaptations, and new programs could potentially be beneficial for strengthening the federal nutrition assistance programs and allowing them to better address food and nutrition security beyond the COVID-19 pandemic. With so many waivers and adaptations, evaluation is needed to understand what was effective, what might be able to be adopted permanently, and what might be useful in future disasters or public health emergencies.

Scoping reviews are a useful tool for examining emerging evidence when there is not enough information or research to address more specific research questions through systematic reviews[12]. They can serve a variety of purposes for studying a particular topic including identifying the type of evidence available, examining how research on the topic has been conducted, and identifying gaps in knowledge [12]. Given that research examining federal food assistance programs during the COVID-19 pandemic is still emerging and the scope of the existing literature is not clear, a scoping review is an appropriate methodology to begin to assess the literature on this topic.

A preliminary search of Pubmed and PROSPERO were conducted and no systematic reviews on federal nutrition assistance programs during the COVID-19 pandemic were published or currently underway were identified. There is one scoping review currently published on the topic, although the goals and scope of this review differ from the proposed scoping review. The currently published review covers the time period of 2000 and 2020, includes other types of emergencies in addition to the COVID-19 pandemic, and focuses largely on lessons learned from the available evidence [13]. While this scoping review makes an important contribution to the literature, there are some gaps that would benefit from an additional scoping review including focusing exclusively on the COVID-19 pandemic, describing the characteristics of the currently published studies (i.e., geographic regions, study population, study methodology); an updated literature search as numerous additional studies have been published related to the topic after 2020; and using more health-focused databases in the literature search.

The goal of this scoping review is to examine the published research on federal nutrition assistance programs during the COVID-19 pandemic, in the U.S., U.S. territories, and tribal nations including programs that were developed during the pandemic (i.e, pandemic electronic benefit transfer). The review will identify the scope of the available research and research and policy recommendations. The results will be shared through two primary products—a peer-reviewed journal article and a public facing document with a bibliography and summary of identified studies.

### Review question

What is the scope of the published research on federal nutrition assistance programs administered by the USDA during the COVID-19 pandemic, including both existing programs (i.e. SNAP, WIC, National School Lunch Program) and new programs that were developed during the pandemic (i.e, P-EBT), in the U.S., U.S. territories, and tribal nations?

#### Aims

1. Describe USDA nutrition assistance programs and program components (e.g., waivers) studied during the COVID-19 pandemic.
2. Map the geographic areas (regions, states, localities) that have been studied related to USDA nutrition assistance programs during the COVID-19 pandemic.
3. Characterize specific subpopulation access and utilization of USDA nutrition assistance programs studied during the COVID-19 pandemic.
4. Describe the outcomes examined in relation to USDA nutrition assistance programs during the COVID-19 pandemic.
5. Identify metrics, indicators, and methodologies used to study USDA nutrition assistance programs during the COVID-19 pandemic.
6. Highlight gaps in the research literature on USDA nutrition assistance programs during the COVID-19 pandemic.

## Materials and methods

The scoping review will be conducted following the guidelines for scoping reviews described by the JBI Manual for Evidence Synthesis [12] and Preferred Reporting Items for Systematic Reviews and Meta-analyses extension for Scoping review (PRISMA-ScR) [12]. The Preferred Reporting Items for Systematic Reviews and Meta-analyses Protocol (PRISMA-P) checklist can be found in S1 Checklist. Institutional review board approval was not required for this study because human subjects were not involved.

### Study eligibility criteria

The eligibility criteria are described below in terms of the population, concept, and context framework [12].

#### Population

The review will include studies focused on the U.S., U.S. territories, and tribal nations.

#### Concept

The review will include studies focused on USDA nutrition assistance programs. This includes the 15 federal nutrition assistance programs administered by the USDA Food and Nutrition Service as well as programs that were developed during the COVID-19 pandemic.

The following programs will be included:

- Supplemental Nutrition Assistance Program (SNAP), Including Disaster SNAP (D-SNAP)
- Special Supplemental Nutrition Program for Women, Infants, and Children (WIC)
- WIC Farmers’ Market Nutrition Program
- Seniors Farmers’ Market Nutrition Program
- Summer Food Service Program (SFSP)
- National School Lunch Program (NSLP)
- School Breakfast Program (SBP)
- Special Milk Program (SMP)
- Fresh Fruit and Vegetable Program (FFVP)
- Child and Adult Care Food Program (CACFP)
- Commodity Supplemental Food Program
- Food Distribution Program on Indian Reservations
- The Emergency Food Assistance Program
- USDA Foods in Schools
- Nutrition Assistance Program (NAP) Block Grants
- Pandemic Electronic Benefit Transfer (P-EBT)
- Farmers to Families Food Box Program
- Emergency Meals to You Program

#### Context

This review will include studies focused on the COVID-19 pandemic.

#### Types of sources

This scoping review will consider both quantitative and qualitative research studies. There will be no limitations on study design and studies using experimental, quasi-experimental, and observational study designs will be included. Only original research articles will be included. Review, text, opinion papers, perspectives, editorials, letters to editors and other non-original research will be excluded. Only studies published in peer-reviewed journals will be included. Published conference abstracts will be excluded. The review will not include grey literature.

### Search strategy

Search terms were identified to help locate articles covering the two main concepts of the research question–Federal nutrition assistance programs administered by the USDA and the COVID-19 pandemic. Both general terms (i.e. food assistance) and specific names for the USDA nutrition assistance programs were included. The search strategy can be found in S1 Appendix.

A librarian was consulted for input on which databases would be most relevant for the topic. The databases to be searched include PubMed, CINHAL, Scopus, and Proquest’s Health Management database. The reference lists of articles meeting the inclusion criteria will be reviewed for additional potentially eligible studies. Included articles will also be searched in google scholar to identify articles that have cited them.

Only studies published in English will be included. Studies published since March 1, 2020 will be included as March 2020 is when the COVID-19 pandemic was considered to be a national emergency in the U.S. and when the USDA began issuing waivers for federal nutrition assistance programs.

### Study/source of evidence selection

References identified through the search will be uploaded into Sciwheel and duplicates will be removed. The deduplicated reference list will be imported into Covidence, a software tool used for conducting systematic reviews, which will be used for title/abstract screening, full-text screening, data abstraction, and quality assessment.

Titles/abstracts will be reviewed by two independent reviewers who will be blinded to each other’s decisions. During this stage, each reviewer will assess the titles and abstracts of articles identified through the literature search and vote yes (the inclusion criteria appear to be met and the article should progress to full-text screening), maybe (not enough information is provided in the title/abstract to decide and the article should progress to full-text screening), or no (the inclusion criteria are not met and the article should not be included). In cases where there is a discrepancy between reviewers, a third reviewer will review the title/abstract to help make a final decision.

Articles included in the full-text screening stage will also be reviewed by two independent reviewers who will be blinded to each other’s decisions with a third reviewer helping make decision in cases where there is a discrepancy between reviewers decisions. During this stage, reviewers will vote as to whether the article meets the inclusion criteria and should be included or if it does not meet the inclusion criteria and should not be excluded. A PRISMA-ScR flow diagram will be included to share information about exclusion reasons and results from the literature and article screening process [14].

### Data extraction

Data will be extracted from each included article by two independent reviewers using a data extraction template in Covidence that will be developed by the study team. The data extraction form will include information on the publication including title, author(s), year of publication, journal, and funding sources. Information gathered through data extraction will include information on programs and program components studied; geographic areas of focus; subpopulations included; outcomes examined; study methodology; and key findings related to the review aims.

The data extraction form will be piloted on a small number of articles. During the pilot testing and data extraction process, the draft data extraction tool will be revised as necessary. Any modifications made will be described in the scoping review. Disagreements that arise between reviewers will be resolved through discussion, or with a third reviewer. If additional information is needed, authors of papers will be contacted to request this information.

### Data analysis and presentation

Extracted data will be reviewed and summarized to address research aims. Characteristics of the studies included and information related to the research aims will be summarized using tables and charts as well as in a narrative summary. The number and percentage of studies with certain characteristics such as particular USDA nutrition assistance programs and program components studied, geographic areas included, and outcomes examined will also be reported.

## Discussion

This scoping review protocol will allow for a comprehensive review of the published peer-reviewed literature on USDA nutrition assistance programs during the COVID-19 pandemic, which has implications for research and policy recommendations. One of the limitations of this protocol is the exclusion of grey literature. While there has been a lot of grey literature published on the topic, we chose to focus exclusively on the scope of what has been published in the peer-reviewed literature for feasibility and because peer-reviewed sources are traditionally viewed as more credible. Only four databases will be searched which may result in articles not indexed in these databases being missed. This scoping review protocol does not include a critical appraisal of studies, as this is an emerging area of research and the main goal of the review is to understand the scope of the published peer-reviewed research as opposed to assessing risk of bias. It is common for scoping reviews not to include critical appraisal of evidence sources [12]. Despite the limitations, this scoping review will make an important contribution to the literature. Because these results may be of interest to multiple audiences, they will be shared through a public facing document with a bibliography and summary of identified studies in addition to a peer-reviewed journal article.

## Supporting information

S1 Checklist PRISMA-P (Preferred Reporting Items for Systematic review and Meta-Analysis Protocols) 2015 checklist: Recommended items to address in a systematic review protocol*. (DOC)

S1 Appendix Search strategy. (DOCX)

## References

[1] U.S. Department of Health and Human Services Office of the Assistant Secretary for Preparedness and Response. Renewal of Determination That A Public Health Emergency Exists [Internet]. 2021 [cited 2021 Dec 12]. Available from: https://www.phe.gov/emergency/news/healthactions/phe/Pages/COVDI-15Oct21.aspx

[2] Executive Office of the President. Declaring a National Emergency Concerning the Novel Coronavirus Disease (COVID-19) Outbreak [Internet]. 2020 [cited 2022 May 11]. Available from: https://www.federalregister.gov/documents/2020/03/18/2020-05794/declaring-a-national-emergency-concerning-the-novel-coronavirus-disease-covid-19-outbreak

[3] Gundersen C, Ziliak JP. Food insecurity and health outcomes. Health Aff (Millwood). 2015 Nov;34(11):1830–9. doi: 10.1377/hlthaff.2015.0645 26526240

[4] Coleman-Jensen A, Rabbitt MP, Gregory CA, Singh A. Household FoodSecurity in the United States in 2020. Washington, DC: U.S. Department of Agriculture, Economic Research Service; 2021. Report No.: ERR-298.

[5] Niles MT, Beavers AW, Clay LA, Dougan MM, Pignotti GA, Rogus S, et al. A Multi-Site Analysis of the Prevalence of Food Insecurity in the United States, before and during the COVID-19 Pandemic. Curr Dev Nutr. 2021 Dec;5(12):nzab135. 10.1093/cdn/nzab135 PMC8677520 34934898

[6] U.S. Department of Agriculture Food and Nutrition Service. FNS Nutrition Programs [Internet]. [cited 2022 Dec 15]. Available from: https://www.fns.usda.gov/programs

[7] Kinsey EW, Hecht AA, Dunn CG, Levi R, Read MA, Smith C, et al. School closures during COVID-19: Opportunities for innovation in meal service. Am J Public Health. 2020 Sep 17;110(11):1635–43. doi: 10.2105/AJPH.2020.305875 32941069 PMC7542295

[8] Lane HG, Turner L, Dunn CG, Hager ER, Fleischhacker S. Leveraging implementation science in the public health response to COVID-19: child food Insecurity and federal nutrition assistance programs. Public Health Rep. 2020 Oct 8;33354920959285. doi: 10.1177/0033354920959285 33031712 PMC7649993

[9] Whaley SE, Anderson CE. The Importance of Federal Waivers and Technology in Ensuring Access to WIC During COVID-19. Am J Public Health. 2021 Jun;111(6):1009–12. doi: 10.2105/AJPH.2021.306211 33856889 PMC8101579

[10] Families First Coronavirus Response Act. 116–127 2020.

[11] U.S. Department of Agriculture Food and Nutrition Service. FNS Responds to COVID-19 [Internet]. [cited 2022 Feb 11]. Available from: https://www.fns.usda.gov/coronavirus

[12] Peters MDJ, McInerney P, Munn Z, Tricco AC, Khalil H. Chapter 11: Scoping Reviews (2020 version). In: Aromataris E, Munn Z, editors. JBI Mannual for Evidence Synthesis. JBI; 2020.

[13] Fraser KT, Shapiro S, Willingham C, Tavarez E, Berg J, Freudenberg N. What we can learn from U.S. food policy response to crises of the last 20 years—Lessons for the COVID-19 era: A scoping review. SSM Popul Health. 2022 Mar;17:100952. 34786449 10.1016/j.ssmph.2021.100952 PMC8582083

[14] Tricco AC, Lillie E, Zarin W, O’Brien KK, Colquhoun H, Levac D, et al. PRISMA Extension for Scoping Reviews (PRISMA-ScR): checklist and explanation. Ann Intern Med. 2018 Oct 2;169(7):467–73. doi: 10.7326/M18-0850 30178033

