# Supplementary material for: United States Department of Agriculture nutrition assistance programs during the COVID-19 pandemic: A scoping review protocol
Source: PLoS One. 2023 Jul 19;18(7):e0288585. doi: 10.1371/journal.pone.0288585 (PMC10355377; doi:10.1371/journal.pone.0288585)
Supplement: S1 Appendix — (DOCX) [file pone.0288585.s002.docx]

**S2 Appendix: Search Strategy**

**PubMed**

*Search Terms:*

(“Supplemental Nutrition Assistance Program”[tiab] OR WIC[tiab] OR “Farmers’ Market Nutrition Program” [tiab] OR “Summer Food Service Program” [tiab] OR “Seamless Summer Option” [tiab] OR “Summer meals” [tiab] OR “Summer nutrition program” [tiab] OR “summer feeding program” [tiab] OR “National School Lunch Program” [tiab] OR “School Breakfast Program”[tiab] OR “School meals” [tiab] OR “Special Milk Program” [tiab] OR “Fresh Fruit and Vegetable Program” [tiab] OR “Child and Adult Care Food Program” [tiab] OR CACFP[tiab] OR “Commodity Supplemental Food Program” [tiab] OR “Food Distribution Program on Indian Reservations” [tiab] OR “Farmers to families food box[tiab]” OR “Food Assistance” [tiab] OR TFAP[tiab] OR USDA[tiab] OR “Nutrition Assistance” [tiab] OR “pandemic electronic benefit transfer[tiab]” OR P-EBT[tiab] OR “nutrition assistance” [tiab] OR “food assistance” [tiab] OR “food benefit*” [tiab] OR “food aid” [tiab] OR “food insecurity” [tiab] OR “hunger” [tiab] OR “food policy” [tiab] OR “nutrition policy” [tiab] OR "Food Assistance"[Mesh])

AND

(COVID-19[tiab] OR Coronavirus[tiab] OR SARS-CoV-2[tiab] OR "COVID-19"[Mesh] OR "SARS-CoV-2"[Mesh] OR "Pandemics"[Mesh])

*Limits*

Published since March 1, 2020; English language

**CINHAL, Scopus, and Proquest’s Health Management database**

*Search Terms*

(“Supplemental Nutrition Assistance Program” OR WIC OR “Farmers’ Market Nutrition Program” OR “Summer Food Service Program” OR “Seamless Summer Option” OR “Summer meals” OR “Summer nutrition program” OR “summer feeding program” OR “National School Lunch Program” OR “School Breakfast Program” OR “School meals” OR “Special Milk Program” OR “Fresh Fruit and Vegetable Program” OR “Child and Adult Care Food Program” OR CACFP OR “Commodity Supplemental Food Program” OR “Food Distribution Program on Indian Reservations” OR “Farmers to families food box” OR “Food Assistance” OR TFAP OR USDA OR “Nutrition Assistance” OR “pandemic electronic benefit transfer” OR P-EBT OR “nutrition assistance” OR “food assistance” OR “food benefit*” OR “food aid” OR “food insecurity” OR “hunger” OR “food policy” OR “nutrition policy” )

AND

(COVID-19 OR Coronavirus OR SARS-CoV-2 OR "SARS-CoV-2")

*CINHAL Limits*

Published since March 2020; English language; Source Type – academic journals

*Scopus Limits*

Published since 2020; English language; Source Type - journal

*Proquest’s Health Management Database Limits*

Published since March 1, 2020; English language; Source Type – scholarly journals
